# Metabolic and endometrial ultrasonographic factors associated with clinical pregnancy in PCOS patients undergoing FET: a nomogram development study with internal validation

**DOI:** 10.3389/fendo.2026.1846250

**Published:** 2026-08-05

**Authors:** Xu Cheng, Linlin Xie, Yitong Zhang, Dan tong, Zhennan Yang, Yuexin Yu, Jing Zhou

**Affiliations:** 1 Department of Reproductive Medicine, General Hospital of Northern Theater Command, Shenyang, Liaoning, China; 2 Department of Outpatient, Liaoning Provincial Corps Hospital, Chinese People's Armed Police Force, Shenyang, Liaoning, China

**Keywords:** clinical pregnancy, frozen-thawed embryo transfer, LASSO regression, metabolic biomarkers, nomogram, polycystic ovary syndrome, predictive model, ultrasonographic parameters

## Abstract

**Objective:**

To explore the independent metabolic and ultrasonographic factors associated with clinical pregnancy in patients with polycystic ovary syndrome (PCOS) undergoing frozen-thawed embryo transfer (FET) cycles, develop and validate a nomogram model for predicting the probability of clinical pregnancy in this population, and offer a preliminary framework for risk stratification that requires external validation.

**Methods:**

We retrospectively enrolled clinical data of PCOS patients who underwent FET in the Reproductive Medicine Department of General Hospital of Northern Theater Command from January 2017 to September 2022. A total of 2316 eligible FET cycles were included and randomly divided into a training cohort (1621 cycles) and an internal validation cohort (695 cycles) at a ratio of 7:3. All cycles were further divided into clinical pregnancy group and non-clinical pregnancy group according to the clinical pregnancy after transplantation. In the training cohort, the least absolute shrinkage and selection operator (LASSO) regression combined with 10-fold cross-validation was used to screen key predictive variables for clinical pregnancy. Variables with non-zero coefficients were included in multivariate logistic regression analysis to identify independent influencing factors, based on which a nomogram prediction model was constructed. The receiver operating characteristic (ROC) curve, calibration curve, Hosmer-Lemeshow goodness-of-fit test, and decision curve analysis (DCA) were performed to verify the discrimination, calibration, and clinical utility of the model.

**Results:**

A total of 2316 FET cycles were included in this study. The clinical pregnancy rate was 63.66% (1032/1621) in the training cohort and 62.16% (432/695) in the internal validation cohort, with no statistically significant difference in baseline data between the two cohorts (P>0.05), indicating good comparability. With lambda.1se as the optimal penalty coefficient, LASSO regression with 10-fold cross-validation finally screened 10 variables with non-zero coefficients that had predictive value for clinical pregnancy, including advanced age, type of infertility, baseline follicle-stimulating hormone (FSH), anti-Müllerian hormone (AMH), total cholesterol (TC), triglycerides (TG), low-density lipoprotein cholesterol (LDL-C), fasting plasma glucose (FPG), endometrial thickness, and number of subendometrial blood flow branches. Multivariate logistic regression analysis showed that secondary infertility, number of subendometrial blood flow branches ≥10, and increased endometrial thickness were independent protective factors for clinical pregnancy in PCOS patients undergoing FET cycles, while elevated levels of TC, TG, LDL-C, and FPG were independent risk factors (P<0.05). The nomogram prediction model was constructed based on the above independent influencing factors. The area under the ROC curve (AUC) of the model was 0.74 (95% CI: 0.72~0.77) in the training cohort and 0.75 (95% CI: 0.72~0.79) in the internal validation cohort. The calibration curve showed good consistency between the predicted probability and the actual clinical pregnancy probability, and the Hosmer-Lemeshow goodness-of-fit test indicated no overfitting of the model. DCA results showed that the model could bring favorable clinical net benefit to patients when the threshold probability was in the range of 0.20 to 0.80.

**Conclusion:**

This internally validated nomogram, incorporating metabolic and ultrasonographic parameters, demonstrated modest discriminative ability for predicting clinical pregnancy in PCOS patients undergoing FET. However, the model explains only a portion of outcome variation, and external validation with additional predictors (including embryo quality and insulin resistance markers) is needed before clinical application.

## 1 Introduction

Polycystic Ovary Syndrome (PCOS) is the most common heterogeneous endocrine and metabolic disorder in women of reproductive age, with a global prevalence of approximately 5% to 10% in this population (1, 2). The core diagnostic features of the disease include oligo-ovulation or anovulation, clinical or biochemical hyperandrogenemia, and polycystic ovarian morphology on ultrasound. It is frequently accompanied by metabolic disorders such as insulin resistance, dyslipidemia, and chronic low-grade inflammation (3–6), as well as reproductive system impairments including impaired endometrial receptivity, reduced oocyte quality, infertility, and an increased risk of adverse clinical pregnancy (7). Compared with healthy women of reproductive age, patients with PCOS have a significantly higher incidence of infertility and are one of the main populations seeking assisted reproductive technology (ART).

*In vitro* fertilization and embryo transfer (IVF-ET) is an effective treatment for infertile patients with PCOS. Frozen-thawed Embryo Transfer (FET) can effectively avoid the risk of ovarian hyperstimulation syndrome (OHSS) caused by supraphysiological doses of estrogen in fresh cycles (8), while improving the synchrony between endometrial development and embryonic growth to enhance endometrial receptivity. At present, it has become one of the preferred ART regimens for patients with PCOS (9, 10). However, in clinical practice, repeated implantation failure still occurs in some PCOS patients during FET cycles. How to accurately assess the pregnancy risk of patients before transplantation and screen modifiable risk factors has become a research hotspot in the field of reproductive medicine.

Clinical prediction models can quantitatively assess the risk of disease occurrence or clinical outcomes by integrating multi-dimensional clinical indicators, and have been widely used in oncology (11–13), cardiovascular disease (14–16), obstetrics and gynecology (17–19), and many other fields (11–19). In recent years, prediction models for ART outcomes in PCOS patients have been gradually developed. However, most existing models only focus on metabolic-related indicators, ignoring the key regulatory role of endometrial receptivity on FET clinical pregnancy, especially lacking models integrating metabolic biomarkers and endometrial ultrasonographic parameters (20, 21). Meanwhile, some models have not undergone comprehensive external validation and clinical utility evaluation, resulting in limited clinical transformation value.

The Least Absolute Shrinkage and Selection Operator (LASSO) regression can compress high-dimensional variables through a penalty function, reducing the coefficients of variables with no predictive value to zero, thus achieving efficient variable screening and effectively avoiding the overfitting problem of traditional methods. It has now become the mainstream method for variable selection in the construction of clinical prediction models. Based on a large sample of clinical data from FET cycles of PCOS patients, this study first screened key predictive variables for clinical pregnancy in PCOS patients undergoing FET cycles through LASSO regression, then integrated patients’ baseline characteristics, metabolic biomarkers, and endometrial receptivity-related ultrasonographic parameters to identify independent influencing factors through multivariate logistic regression, and finally constructed and validated a nomogram prediction model. The purpose of this study is to explore the potential feasibility of a quantitative risk assessment approach to identify candidate predictors for future, more comprehensive models. We acknowledge that this is an exploratory, single-center study; findings should be interpreted as hypothesis-generating rather than practice-changing.

## 2 Materials and methods

### 2.1 Study population

We retrospectively enrolled the clinical data of PCOS patients who underwent FET in the Reproductive Medicine Department of General Hospital of Northern Theater Command from January 2017 to September 2022. This study was approved by the Medical Ethics Committee of General Hospital of Northern Theater Command (Ethics Approval No. 2020PJ010), and all study procedures strictly followed the ethical principles of the *Declaration of Helsinki*. Given the retrospective nature of this study, informed consent was waived by the ethics committee; all clinical data of patients were de-identified to strictly protect patient privacy.

Inclusion criteria: Patients met the 2003 Rotterdam diagnostic criteria for PCOS (22), which requires the presence of at least two of the following three items: ① oligo-ovulation or anovulation; ② clinical manifestations of hyperandrogenism (hirsutism, acne, etc.) or biochemical hyperandrogenemia; ③ transvaginal ultrasound indicating polycystic ovarian morphology (≥12 antral follicles with a diameter of 2–9 mm in unilateral ovary, and/or ovarian volume >10 mL; ovarian volume = 0.5 × length × width × thickness, unit: cm³). All patients underwent FET in our center with high-quality embryos (cleavage-stage embryos or blastocysts) and complete clinical data.

Exclusion criteria: ① Complicated with other diseases that may cause menstrual disorders or hyperandrogenemia, including Cushing’s syndrome, congenital adrenal hyperplasia, and thyroid dysfunction; ② Complicated with uterine organic lesions such as uterine malformation, moderate to severe endometriosis, and intrauterine adhesions; ③ Chromosomal abnormalities in either partner; ④ Severe male infertility factors in the male partner, including severe oligoasthenoteratozoospermia and non-obstructive azoospermia; ⑤ Preimplantation genetic testing (PGT) performed in this cycle; ⑥ Repeated FET cycles, only data from the first FET cycle of the patient were included; ⑦ Incomplete clinical data, loss to follow-up, or failure to complete follow-up of clinical pregnancy in this cycle.

After excluding cycles with incomplete clinical data (missing rate for key variables approximately 1%), 2,316 cycles were eligible for final analysis. Comparison of baseline characteristics between excluded and included patients showed no significant differences (all *P* > 0.05), indicating that data were missing completely at random. Complete-case analysis was performed, and given the extremely low missing proportion, the impact on result robustness is negligible. A total of 2316 eligible first FET cycles were finally included, and randomly divided into a training cohort (1621 cycles) and an internal validation cohort (695 cycles) using a random number table method at a ratio of 7:3. The training cohort was used for variable screening, identification of independent influencing factors, and construction of the nomogram model, while the internal validation cohort was used for model validation to evaluate the generalization ability of the model.

### 2.2 Outcome measures

The clinical data of all patients were collected through the hospital electronic medical record system, including the following four categories of indicators:

1. Demographic characteristics: including female age, body mass index (BMI), duration of infertility, type of infertility, baseline FSH, and anti-Müllerian hormone (AMH). BMI ≥28 kg/m² was defined as obesity, and age ≥35 years was defined as advanced age.
2. Metabolic biomarkers: including fasting total cholesterol (TC), triglycerides (TG), low-density lipoprotein cholesterol (LDL-C), and fasting plasma glucose (FPG) tested within 3 months before transplantation.
3. FET cycle-related parameters: endometrial preparation protocol (artificial cycle/natural cycle), type of embryos transferred (cleavage-stage embryo/blastocyst), and number of embryos transferred.
4. Ultrasonographic parameters: endometrial thickness, endometrial pattern (Type A/non-Type A), and number of subendometrial blood flow branches detected by transvaginal ultrasound on the day of transplantation. All the ultrasound examinations in this study were conducted by three senior physicians with over 5 years of experience in reproductive ultrasound. They uniformly used the same type of ultrasound equipment (GE Voluson E8, GE Healthcare, USA) and followed a standardized operation protocol. Before the study began, all the ultrasound physicians received unified training (23): the patient was placed in the lithotomy position with calm breathing, and the double-layer endometrial thickness was measured in the median sagittal section of the uterus (24). Endometrial pattern was evaluated according to the Gonen criteria (25): Type A was defined as a triple-line endometrium, and non-Type A included homogeneous and blurred endometrium. The power Doppler flow imaging mode was activated with a pulse repetition frequency of 0.6 MHz to observe and record the number of subendometrial blood flow branches, which were grouped according to the criteria of relevant studies (26): Group A (≤7 branches), Group B (8~9 branches), and Group C (≥10 branches).

### 2.3 Endometrial preparation protocols for FET cycles

Two main endometrial preparation protocols were used in the FET cycles of this study: natural cycle and artificial cycle (hormone replacement cycle), with specific procedures as follows:

Natural cycle protocol: Applicable to patients with regular menstrual cycles and spontaneous ovulation. Ultrasound examination was performed on day 3–5 of the menstrual cycle to rule out ovarian cysts, and follicular development was monitored by ultrasound from day 10–12 of the menstrual cycle. When the follicle diameter reached 16–20 mm, serum estradiol, progesterone, and luteinizing hormone levels were measured to determine the ovulation date. Thawed embryo transfer was performed 3–5 days after ovulation.

Artificial cycle protocol: Applicable to patients with irregular menstrual cycles and anovulation. Endometrial thickness was monitored by ultrasound on day 3–5 of the menstrual cycle, and estradiol valerate 2 mg twice daily was administered on the same day. The dose was adjusted every 4 days according to the endometrial thickness. After 12–14 days, ultrasound examination was performed and serum progesterone level was determined. When the endometrial thickness reached 6 mm, intramuscular injection of progesterone 40 mg/day and oral allylestrenol 10 mg were administered and maintained until embryo transfer. Cleavage-stage embryos or blastocysts were transferred 3–5 days after endometrial transformation. Luteal support was continued after embryo transfer until 10 weeks of gestation.

### 2.4 Embryo culture and transfer

Cleavage-stage embryos were graded using the Veeck classification: high-quality cleavage embryos were defined as grade 1–2 on day 3 with 7–9 blastomeres and fragmentation rate <20%. Blastocysts were graded using the Gardner classification: high-quality blastocysts were defined as day 5–6 blastocysts with inner cell mass and trophectoderm score A or B (27, 28). Single or double embryo transfer was performed after thawing, determined by clinicians. Luteal support was continued after embryo transfer; serum β-human chorionic gonadotropin (β-HCG) positive patients received sustained support until 8–10 weeks of gestation with gradual tapering. We only included morphologically high-quality embryos in all cycles to reduce confounding bias, yet standardized molecular embryo metrics were not available for model construction, representing a significant limitation of this study.

### 2.5 Outcome definition

Serum human chorionic gonadotropin (β-HCG) was measured 14 days after transplantation. If β-HCG was positive, transvaginal ultrasound was performed 28–30 days after embryo transfer. Clinical pregnancy was defined as the presence of an intrauterine gestational sac on ultrasound.

### 2.6 Statistical analysis

The Shapiro-Wilk test was used to test the normality of measurement data. Normally distributed measurement data were expressed as mean ± standard deviation (x ± s), and the independent samples t-test was used for intergroup comparison. Non-normally distributed measurement data were expressed as median (interquartile range) [M (Q1, Q3)], and the Mann-Whitney U test was used for intergroup comparison. Categorical data were expressed as number (percentage) [n (%)], and the chi-square test or Fisher’s exact test was used for intergroup comparison.

Variable screening: In the training cohort, with clinical pregnancy as the dependent variable and all clinical indicators as independent variables, LASSO regression was performed for variable screening using the “glmnet” package (version 4.1-8) of R software (version 4.3.3, alpha=1). The optimal penalty coefficient λ was determined by 10-fold cross-validation, and lambda.1se (the maximum λ value that keeps the model error within one standard error of the minimum error) was selected as the optimal value. Variables with non-zero regression coefficients were retained and included in the subsequent multivariate logistic regression analysis.

Screening of independent influencing factors: Variables with non-zero coefficients screened by LASSO regression were included in the multivariate logistic regression model to identify independent influencing factors for clinical pregnancy in PCOS patients undergoing FET cycles, and the odds ratio (OR) and 95% confidence interval (CI) were calculated. Multicollinearity test was performed for all variables included in the analysis, with tolerance <0.5 or variance inflation factor (VIF) >5 defined as the criteria for severe multicollinearity.

Model construction and validation: The nomogram prediction model was constructed based on the independent influencing factors screened by multivariate regression analysis, and Bootstrap resampling (1000 repetitions) was used for internal validation. The AUC of the ROC curve was calculated to evaluate the discrimination of the model; the consistency between the predicted probability of the model and the actual outcome was evaluated by the calibration curve and Hosmer-Lemeshow goodness-of-fit test; the clinical net benefit and utility of the model were evaluated by DCA using the “rmda” package (version 1.6.3) of R software. Missing data were handled using complete-case analysis, as the proportion of missing data for key variables was <2%. Baseline characteristics were comparable between included and excluded patients, suggesting that the missing data were unlikely to introduce systematic bias.

All statistical analyses were performed using R 4.3.3 software, and P<0.05 was considered statistically significant.

## 3 Results

### 3.1 Comparison of baseline data between training and internal validation cohorts

A total of 2316 first FET cycles of PCOS patients were included in this study, including 1621 cycles in the training cohort and 695 cycles in the internal validation cohort. There were no statistically significant differences in age, BMI, baseline endocrine indicators, metabolic indicators, FET cycle parameters, ultrasonographic indicators, and clinical pregnancy rate between the two cohorts (P>0.05), indicating good comparability (**Table 1**). The clinical pregnancy rate was 63.66% (1032/1621) in the training cohort and 62.16% (432/695) in the internal validation cohort.

**Table 1:** Characteristics of the training and internal validation cohorts(N=2316).

Variables	Training cohort (n = 1621)	Validation cohort (n = 695)	P-value
Female age (years)	32.00 (29.00, 34.00)	32.00 (30.00, 34.00)	0.981
Advanced Age, n (%)			0.928
<35	1231 (75.94)	529 (76.12)	
≥35	390 (24.06)	166 (23.88)	
BMI (kg/m2)	25.10 (22.00, 28.10)	24.80 (21.80, 27.95)	0.147
Obesity, n (%)			0.784
No	1197 (73.84)	517 (74.39)	
Yes	424 (26.16)	178 (25.61)	
AMH (ng/ml)	6.17 (4.25, 8.34)	6.10 (4.25, 8.34)	0.951
Baseline FSH (IU/L)	5.20 (4.31, 6.16)	5.15 (4.23, 6.12)	0.343
Duration of infertility (years)	3.00 (2.00, 5.00)	3.00 (2.00, 5.00)	0.257
Type of infertility, n (%)			0.695
Primary infertility	889 (54.84)	375 (53.96)	
Secondary infertility	732 (45.16)	320 (46.04)	
FET protocols, n (%)			0.397
Artificial cycle	1431 (88.28)	622 (89.50)	
Natural cycle	190 (11.72)	73 (10.50)	
Type of embryos transferred, n (%)			0.142
Cleavage embryo	430 (26.53)	205 (29.50)	
Blastocyst	1191 (73.47)	490 (70.50)	
No. of embryos transferred, n (%)			0.120
1	752 (46.39)	298 (42.88)	
2	869 (53.61)	397 (57.12)	
TC (mmol/L)	4.33 (3.35, 4.70)	4.33 (3.40, 4.73)	0.592
TG (mmol/L)	1.36 (0.99, 1.90)	1.40 (1.00, 1.90)	0.910
LDL-C (mmol/L)	2.79 (2.20, 3.22)	2.77 (2.20, 3.30)	0.365
FPG(mmol/L)	5.15 (4.86, 5.52)	5.14 (4.83, 5.49)	0.798
Endometrial thickness (cm)	0.98 (0.82, 1.10)	0.96 (0.82, 1.10)	0.407
Endometrial pattern, n (%)			0.280
Type A	422 (26.03)	196 (28.20)	
Type non-A	1199 (73.97)	499 (71.80)	
No. of subendometrial blood flow branches, n (%)			0.733
A	492 (30.35)	222 (31.94)	
B	772 (47.62)	326 (46.91)	
C	357 (22.02)	147 (21.15)	
Pregnancy outcomes, n (%)			0.491
Non-pregnancy	589 (36.34)	263 (37.84)	
Pregnancy	1032 (63.66)	432 (62.16)	

Data are shown as median (interquartile range), or no. (%).

BMI, body mass index; AMH, AntiMullerian hormone; FSH, follicular stimulating hormone, TC, total cholesterol, TG, triglycerides, LDL-C, low-density lipoprotein cholesterol, FPG, fasting plasma glucose.

### 3.2 Comparison of baseline data between clinical pregnancy and non-clinical pregnancy groups in the training cohort

The 1621 cycles in the training cohort were divided into clinical pregnancy group (1032 cases) and non-clinical pregnancy group (589 cases) according to whether clinical pregnancy was achieved. The comparison of baseline data between the two groups showed that: patients in the clinical pregnancy group had significantly lower age, duration of infertility, proportion of advanced age, proportion of obesity, baseline FSH, TC, TG, and LDL-C levels than those in the non-clinical pregnancy group (P<0.05); while the proportion of blastocyst transfer, endometrial thickness, proportion of Type A endometrium, and proportion of Group C in terms of the number of subendometrial blood flow branches were significantly higher than those in the non-clinical pregnancy group (P<0.05) (**Table 2**).

**Table 2:** Baseline characteristics according to the pregnancy outcomes in the training cohort (N=1621).

Variables	Clinical pregnancy (n = 1020)	Non-clinical pregnancy (n = 601)	*P*-value
Female age (years)	32.00 (29.00, 34.00)	32.00 (30.00, 35.00)	**<0.001**
Advanced age, n(%)			<0.001
<35	811 (79.51)	384 (63.89)	
≥35	209 (20.49)	217 (36.11)	
BMI (kg/m^2^)	25.00 (22.00, 28.00)	25.40 (22.00, 28.20)	0.314
Duration of infertility (years)	3.00 (2.00, 5.00)	3.00 (2.00, 6.00)	**0.016**
Obesity, n(%)			<0.001
No	792 (77.65)	404 (67.22)	
yes	228 (22.35)	197 (32.78)	
AMH (ng/ml)	6.34 (4.35, 8.53)	5.90 (4.16, 8.62)	0.241
Baseline FSH (IU/L)	5.16 (4.19, 6.05)	5.31 (4.40, 6.20)	**0.046**
Type of infertility, n(%)			0.497
Primary infertility	544 (53.33)	331 (55.07)	
Secondary infertility	476 (46.67)	270 (44.93)	
FET protocols, n(%)			0.260
Artificial cycle	913 (89.51)	527 (87.69)	
Natural cycle	107 (10.49)	74 (12.31)	
Type of embryos transferred, n(%)			<0.001
Cleavage embryo	250 (24.51)	212 (35.27)	
Blastocyst	770 (75.49)	389 (64.73)	
No. of embryos transferred, n(%)			0.261
1	474 (46.47)	262 (43.59)	
2	546 (53.53)	339 (56.41)	
TC (mmol/L)	3.92 (3.30, 4.64)	4.70 (4.29, 4.77)	**<0.001**
TG (mmol/L)	1.20 (0.96, 1.52)	1.90 (1.04, 1.90)	**<0.001**
LDL-C (mmol/L)	2.50 (2.07, 3.07)	3.01 (2.68, 3.30)	**<0.001**
FPG (mmol/L)	5.12 (4.85, 5.48)	5.17 (4.88, 5.60)	0.194
Endometrial thickness (cm)	1.00 (0.85, 1.12)	0.91 (0.80, 1.10)	**<0.001**
Endometrial pattern, n (%)			0.025
Type A	293 (28.73)	142 (23.63)	
Type non-A	727 (71.27)	459 (76.37)	
No. of Sub-endometrial blood, n (%)			<0.001
A	250 (24.51)	241 (40.10)	
B	482 (47.25)	306 (50.92)	
C	288 (28.24)	54 (8.99)	

Data are shown as means ± SD, median (interquartile range), or no. (%).

BMI, body mass index; AMH, AntiMullerian hormone; FSH, follicular stimulating hormone, TC, total cholesterol; TG, triglycerides; LDL-C, low-density lipoprotein cholesterol;s FPG, fasting plasma glucose. Bold values indicate statistically significant differences (P < 0.05).

### 3.3 Feature selection and parameter construction

**Figure 1:**
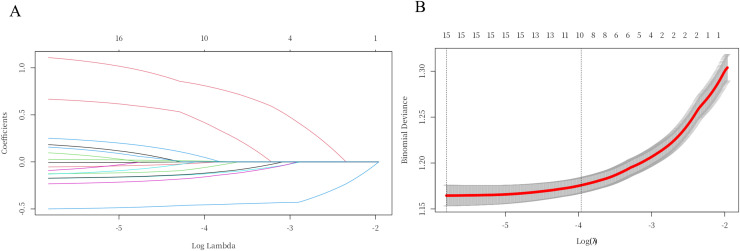
Variable selection via least absolute shrinkage and selection operator (LASSO) regression. Panel **(A)** shows the cross-validation curve of LASSO regression. The x-axis represents the log-transformed value of the penalty coefficient λ (logλ), and the y-axis represents the cross-validation error (mean squared error). The red dashed line corresponds to the optimal penalty coefficient lambda.1se=0.02045 (the maximum λ value that keeps the model error within one standard error of the minimum error). Panel **(B)** shows the variable coefficient trajectory plot, where each curve represents one candidate variable. As the λ value increases, the variable coefficients are gradually compressed. Finally, 10 variables retain non-zero coefficients (advanced age, type of infertility, baseline FSH, AMH, TC, TG, LDL-C, FPG, endometrial thickness, and number of subendometrial blood flow branches), while the coefficients of the remaining 6 variables (duration of infertility, BMI, endometrial preparation protocol, endometrial pattern, type of embryos transferred, number of embryos transferred) are compressed to 0 and excluded. This screening was completed by 10-fold cross-validation to ensure the stability and reliability of variable selection.

LASSO binary logistic regression analysis was performed with clinical pregnancy in the training cohort as the dependent variable and candidate clinical indicators as independent variables, and the optimal penalty coefficient λ was determined by 10-fold cross-validation. The results showed that when λ=lambda.1se (0.02045), the model fitting error was within one standard error of the minimum error, and the number of variables was optimal (**Figure 1**). At this point, 10 variables had non-zero regression coefficients, namely advanced age, type of infertility, baseline FSH, AMH, TC, TG, LDL-C, FPG, endometrial thickness, and number of subendometrial blood flow branches.

The regression coefficients of the remaining 6 variables (duration of infertility, BMI, endometrial preparation protocol, endometrial pattern, type of embryos transferred, number of embryos transferred) were compressed to 0 in the LASSO model, and thus were not included in the subsequent multivariate regression analysis. This does not mean that these variables have no clinical value; for example, the exclusion of BMI is most likely due to its collinearity with metabolic indicators, which will be further discussed in the Discussion section. (**Figure 1B**).

### 3.4 Results of multivariate logistic regression analysis

The 10 variables with non-zero coefficients screened by LASSO regression were included in the backward stepwise multivariate logistic regression model. The multicollinearity test showed that the tolerance of all variables was >0.5 and VIF was <5, indicating no severe multicollinearity. The results showed (**Table 3**): secondary infertility (OR = 1.35, 95% CI: 1.08~1.70, P = 0.009), Group C of the number of subendometrial blood flow branches (≥10 branches, OR = 3.35, 95% CI: 2.26~4.98, P<0.001), and increased endometrial thickness (OR = 1.93, 95% CI: 1.03~3.59, P = 0.039) were independent protective factors for clinical pregnancy in PCOS patients undergoing FET cycles; while elevated TC (OR = 0.62, 95% CI: 0.48~0.80, P<0.001), elevated TG (OR = 0.81, 95% CI: 0.71~0.93, P = 0.002), elevated LDL-C (OR = 0.76, 95% CI: 0.58~0.99, P = 0.045), and elevated FPG (OR = 0.82, 95% CI: 0.72~0.93, P = 0.002) were independent risk factors. Elevated baseline FSH (OR = 0.94, 95% CI: 0.87~1.01, P = 0.102) and elevated AMH (OR = 1.03, 95% CI: 1.00~1.06, P = 0.082) showed borderline statistical significance.

**Table 3:** The univariate and multivariate logistic regression analysis for factors associated pregnancy outcomes in the training cohort (N=1621).

	Univariable		Multivariable	
Variables	OR (95% CI)	*P* value	OR (95% CI)	*P* value
Advanced age, n(%)				
<35	1.00 (Reference)		1.00 (Reference)	
≥35	0.46 (0.36 ~ 0.57)	**<0.001**	0.65 (0.50 ~ 0.84)	**0.001**
Type of infertility, n(%)				
Primary infertility	1.00 (Reference)		1.00 (Reference)	
Secondary infertility	1.07 (0.88 ~ 1.31)	0.497	1.41 (1.12 ~ 1.78)	**0.004**
Obesity, n(%)				
No	1.00 (Reference)		1.00 (Reference)	
yes	0.59 (0.47 ~ 0.74)	**<0.001**	0.68 (0.52 ~ 0.88)	**0.004**
FET protocols, n(%)				
Artificial cycle	1.00 (Reference)			
Natural cycle	0.83 (0.61 ~ 1.14)	0.261		
Endometrial pattern, n (%)				
Type A	1.00 (Reference)			
Type non-A	0.77 (0.61 ~ 0.97)	**0.025**		
No. of Sub-endometrial blood, n (%)				
A	1.00 (Reference)		1.00 (Reference)	
B	1.52 (1.21 ~ 1.91)	**<0.001**	1.68 (1.29 ~ 2.18)	**<0.001**
C	5.14 (3.66 ~ 7.23)	**<0.001**	4.59 (3.15 ~ 6.67)	**<0.001**
Type of embryos transferred, n(%)				
Cleavage embryo	1.00 (Reference)		1.00 (Reference)	
Blastocyst	1.68 (1.35 ~ 2.09)	**<0.001**	1.34 (1.04 ~ 1.72)	**0.021**
No. of embryos transferred, n(%)				
1	1.00 (Reference)			
2	0.89 (0.73 ~ 1.09)	0.261		
Duration of infertility (years)	0.95 (0.91 ~ 0.98)	**0.003**		
TC (mmol/L)	0.42 (0.37 ~ 0.48)	**<0.001**	0.48 (0.41 ~ 0.56)	**<0.001**
TG (mmol/L)	0.60 (0.53 ~ 0.68)	**<0.001**	0.78 (0.68 ~ 0.91)	**<0.001**
LDL-C (mmol/L)	0.45 (0.39 ~ 0.53)	**<0.001**		
FPG (mmol/L)	0.88 (0.78 ~ 0.99)	**0.029**		
Endometrial thickness (cm)	3.22 (1.95 ~ 5.30)	**<0.001**		
Baseline FSH (IU/L)	0.93 (0.87 ~ 0.99)	**0.028**	0.93 (0.87 ~ 1.00)	0.064
AMH (ng/ml)	1.00 (0.98 ~ 1.03)	0.720		

Data are as odds ratio (95% CI), P value.

BMI, body mass index; AMH, AntiMullerian hormone; FSH, follicular stimulating hormone, TC, total cholesterol; TG, triglycerides; LDL-C, low-density lipoprotein cholesterol; FPG, fasting plasma glucose. Bold values indicate statistically significant differences (P < 0.05).

### 3.5 Construction and validation of the nomogram prediction model

#### 3.5.1 Model construction

**Figure 2:**
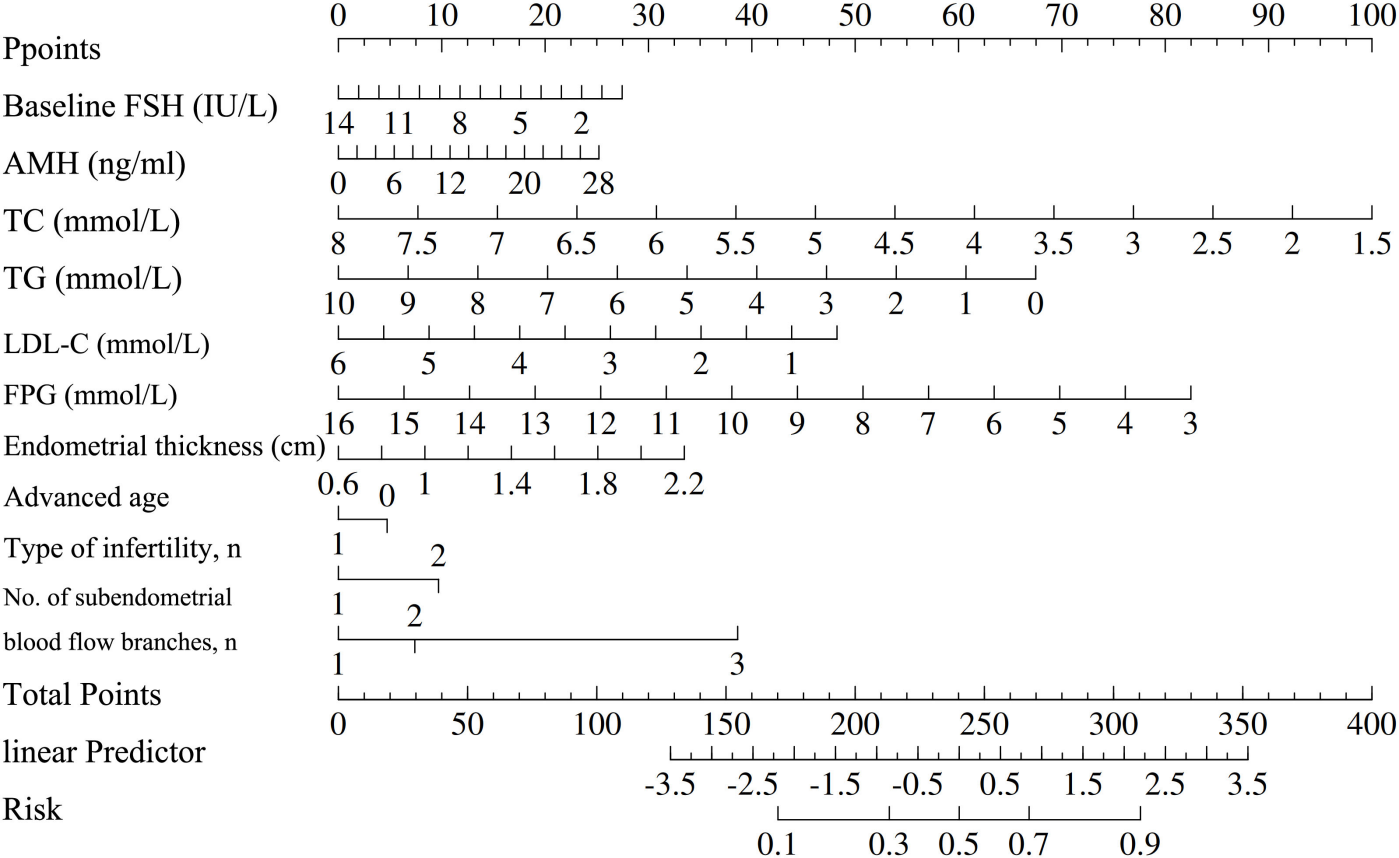
Nomogram for predicting clinical pregnancy probability in PCOS patients undergoing FET cycles. This nomogram was constructed based on 7 independent influencing factors (type of infertility, number of subendometrial blood flow branches, endometrial thickness, TC, TG, LDL-C, FPG) and 2 borderline significant indicators (baseline FSH, AMH). Usage instructions: ① Find the corresponding value of the patient on each variable axis; ② Draw a vertical line upward from this value to the “Points” axis to obtain the single score of the variable; ③ Accumulate the single scores of all variables to obtain the “Total Points”; ④ Draw a vertical line downward from the total score to the “Risk” axis to read the personalized predicted probability of clinical pregnancy in the FET cycle of the patient. The model is easy to operate and can quickly provide quantitative risk assessment for clinical practice.

Based on the 7 independent influencing factors screened by multivariate logistic regression analysis (type of infertility, number of subendometrial blood flow branches, endometrial thickness, TC, TG, LDL-C, FPG), we further retained baseline FSH and AMH with borderline statistical significance (P = 0.102 and P = 0.082, respectively) to construct the final nomogram model (**Figure 2**). This decision was supported by both clinical and methodological considerations: (1) Clinically, FSH and AMH are well-recognized core markers of ovarian reserve and established prognostic factors for ART outcomes, and their exclusion based solely on P values would reduce the clinical interpretability and biological plausibility of the model. (2) Methodologically, both variables were selected by LASSO regression with 10-fold cross-validation and retained non-zero coefficients, indicating they contribute to the overall predictive performance of the model.

#### 3.5.2 Clinical example of nomogram use

To illustrate the clinical application of the nomogram, we present a hypothetical case. A 31-year-old PCOS patient (not advanced age, score 0) with secondary infertility (score 20), endometrial thickness of 1.1 cm (score 28), subendometrial blood flow Group C (≥10 branches, score 50), TC 3.5 mmol/L (score 22), TG 1.2 mmol/L (score 18), LDL-C 2.5 mmol/L (score 20), FPG 5.0 mmol/L (score 20), FSH 4.5 IU/L (score 22), and AMH 5.5 ng/mL (score 15). The total score is 215, corresponding to a predicted clinical pregnancy probability of approximately 78%. In contrast, a second patient with similar baseline characteristics but subendometrial blood flow Group A (≤7 branches, score 0), TC 4.8 mmol/L, TG 1.9 mmol/L, and LDL-C 3.2 mmol/L would have a total score of approximately 145, corresponding to a predicted probability of approximately 45%. This example demonstrates how the nomogram can assist in identifying patients at higher risk of non-pregnancy who may benefit from pre-transfer interventions such as lifestyle modification or metabolic optimization, although prospective studies are needed to confirm whether such interventions actually improve outcomes in these high-risk patients.

#### 3.5.3 Discrimination validation of the model

**Figure 3:**
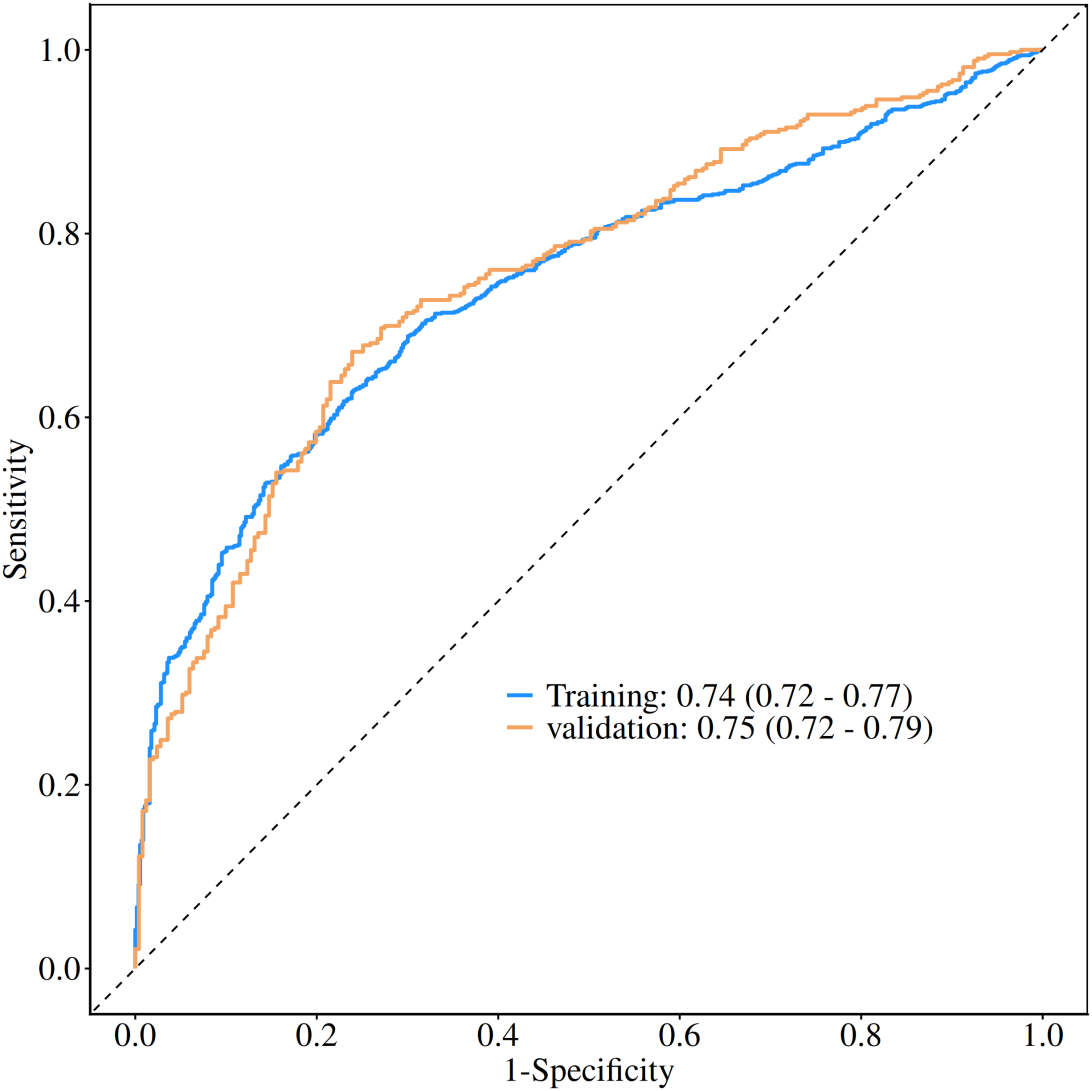
Receiver operating characteristic (ROC) curves of the training and internal validation sets. The blue curve represents the training cohort (n=1621), and the red curve represents the internal validation cohort (n=695). The x-axis is the false positive rate (1 - specificity), and the y-axis is the true positive rate (sensitivity). The area under the ROC curve (AUC) is 0.74 (95% confidence interval CI: 0.72~0.77) for the training cohort and 0.75 (95% CI: 0.72~0.79) for the internal validation cohort, with an optimal cut-off value of 0.6765 for both cohorts. The results indicate that the nomogram model has acceptable discrimination in both the training and internal validation cohorts, and can effectively distinguish between pregnant and non-pregnant patients.

ROC curve analysis showed that the AUC of the nomogram model was 0.74 (95% CI: 0.72~0.77) in the training cohort, with an optimal cut-off value of 0.6765, corresponding to a sensitivity of 0.63 (0.60~0.66), a specificity of 0.76 (0.72~0.79), a positive predictive value (PPV) of 0.82 (0.80~0.85), and a negative predictive value (NPV) of 0.53 (0.50~0.57). In the internal validation cohort, the AUC was 0.75 (95% CI: 0.72~0.79), with the same optimal cut-off value of 0.6765, corresponding to a sensitivity of 0.64 (0.59~0.68), a specificity of 0.78 (0.73~0.83), a PPV of 0.83 (0.79~0.87), and an NPV of 0.56 (0.51~0.61). These results indicated that the model had acceptable discrimination(**Figure 3**; **Table 4**). The AUC of 0.74-0.75 indicates that approximately 75-80% of outcome variation remains unexplained by the current predictor set.

**Table 4:** Predictive performance of the nomogram for clinical pregnancy outcomes.

Indicators	Training set	Validation set
AUC(95%CI)	0.74 (0.72~0.77)	0.75 (0.72~0.79)
Cutoff value	0.6765	0.6765
Sensitivity (%)	0.63 (0.60 ~ 0.66)	0.64 (0.59 ~ 0.68)
Specificity (%)	0.76 (0.72 ~ 0.79)	0.78 (0.73 ~ 0.83)
PPV (%)	0.82 (0.80 ~ 0.85)	0.83 (0.79 ~ 0.87)
NPV (%)	0.53 (0.50 ~ 0.57)	0.56 (0.51 ~ 0.61)

AUC, Area under the curve; PPV, Positive predictive value; NPV, Negative predictive value.

The optimal cut-off value was determined by maximizing the Youden’s index.

#### 3.5.4 Calibration validation of the model

**Figure 4:**
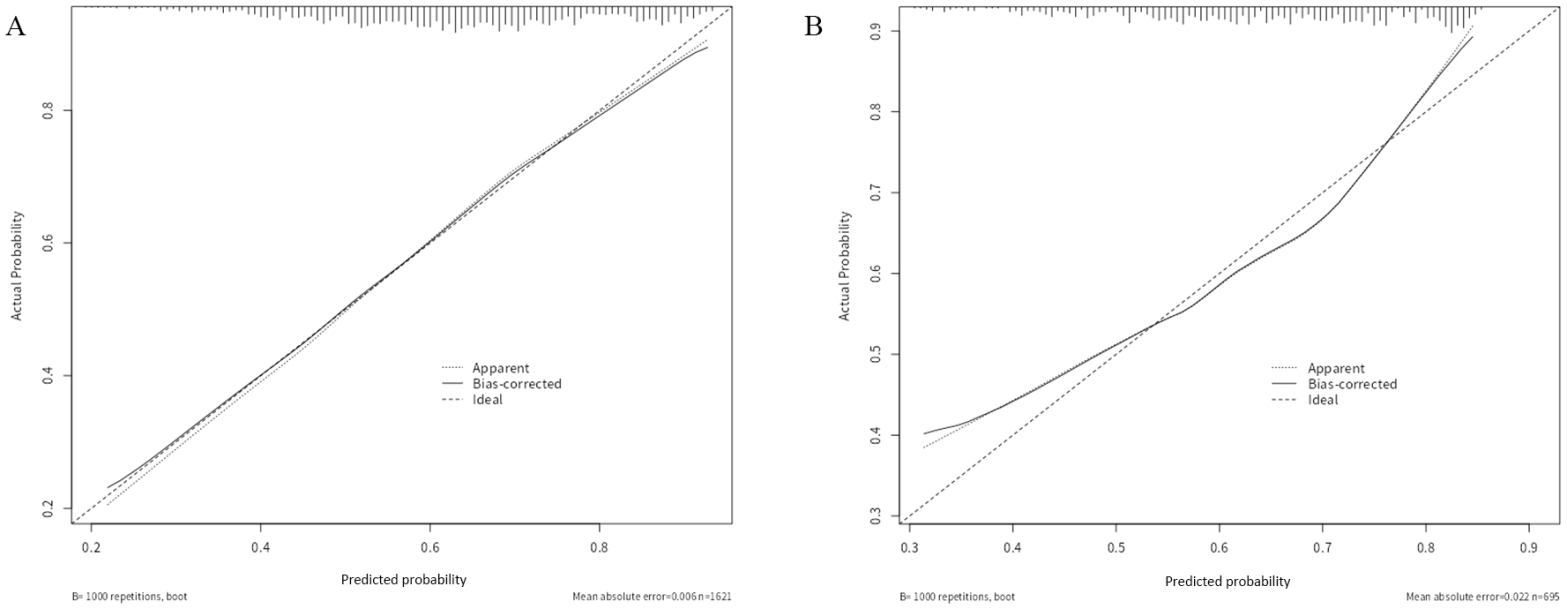
Calibration curves of the training and internal validation sets. Panel **(A)** is the training cohort, and Panel **(B)** is the internal validation cohort. The black dashed line is the ideal calibration curve (predicted probability = actual probability), and the red solid line is the model calibration curve (corrected by 1000 times of Bootstrap resampling). The Hosmer-Lemeshow goodness-of-fit test showed χ²=4.32, P = 0.83 for the training cohort, and χ²=6.18, P = 0.63 for the internal validation cohort, with no statistically significant differences. The mean absolute error was 0.006 in the training cohort and 0.022 in the internal validation cohort. The calibration curve is in good agreement with the ideal curve, indicating that the clinical pregnancy probability predicted by the model is highly consistent with the actual pregnancy probability, without overfitting, and has excellent calibration accuracy.

The calibration curve results showed that after 1000 times of Bootstrap resampling correction, the calibration curves of the model in both the training and internal validation cohorts were in good agreement with the ideal diagonal line (predicted probability = actual probability) (**Figure 4**), indicating a high consistency between the clinical pregnancy probability predicted by the model and the actual clinical pregnancy probability. The Hosmer-Lemeshow goodness-of-fit test showed that χ²=4.32, P = 0.83 for the training cohort, and χ²=6.18, P = 0.63 for the internal validation cohort, with no statistically significant differences, indicating that the model had a good fitting effect without overfitting. The mean absolute error was 0.006 in the training cohort and 0.022 in the internal validation cohort, which further confirmed the excellent calibration accuracy of the model.

#### 3.5.5 Clinical utility validation of the model

**Figure 5:**
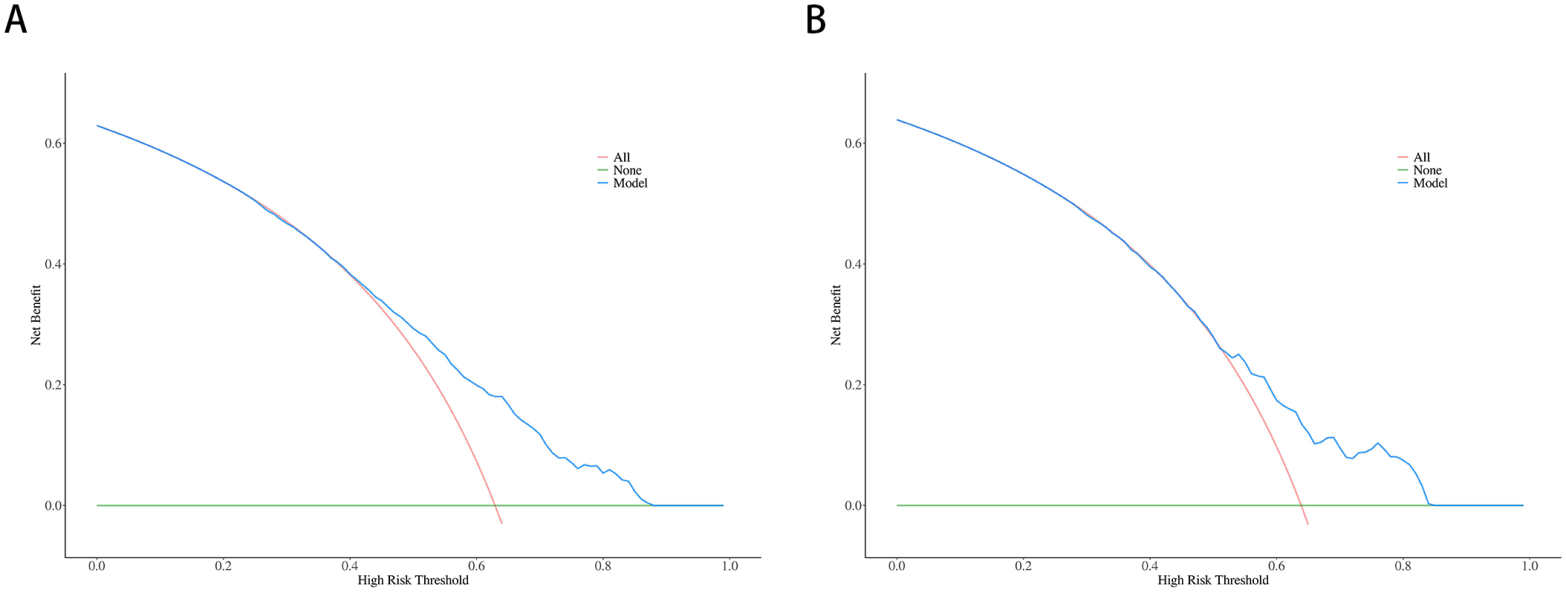
Decision curve analysis (DCA) of the training and internal validation sets. Panel **(A)** is the training cohort, and Panel **(B)** is the internal validation cohort. The x-axis is the threshold probability, which represents the minimum pregnancy probability threshold for clinicians to judge that a patient needs transplantation intervention. The y-axis is the net benefit, which is the benefit brought by the model-guided clinical decision minus the loss of false positives and false negatives. The blue curve is the nomogram model, the red curve is the “all intervention” strategy (all patients undergo embryo transfer), and the green curve is the “no intervention” strategy (none of the patients undergo embryo transfer). The results show that when the threshold probability is in the range of 0.20~0.80, the net benefit of the nomogram model is significantly higher than that of the “all intervention” and “no intervention” strategies, indicating that the model only provides auxiliary risk reference within the threshold range, and formal clinical application still requires prospective external verification.

DCA results showed that when the threshold probability was in the range of 0.20~0.80, the net benefit of the nomogram model was significantly higher than that of the “all intervention” (all patients underwent transplantation) and “no intervention” (none of the patients underwent transplantation) strategies (**Figure 5**). Application of this model for clinical pregnancy risk assessment within this threshold range can bring favorable clinical net benefit to patients, indicating that the model has high clinical utility and may offer supplemental information for risk discussion, though prospective validation is required.

## 4 Discussion

Since the first successful delivery via frozen-thawed embryo transfer was reported by Trounson et al. in 1983, FET has become a core component of ART. FET can not only effectively improve the cumulative pregnancy rate of ART and reduce the risk of OHSS, but also flexibly adjust the transplantation time when endometrial receptivity is poor, providing more possibilities for improving reproductive outcomes. As a recurrent endocrine and metabolic disease, PCOS is characterized by excessive secretion of androgens and luteinizing hormone, often accompanied by elevated levels of inflammatory factors, decreased expression of avβ3 integrin and glycated proteins, which further lead to impaired endometrial receptivity, reduced fertility, and impaired oocyte maturation and embryo quality. With the development of embryo vitrification technology, FET regimens have been widely used in IVF/ICSI treatment of PCOS patients. This regimen can not only reduce the risk of OHSS, but also reduce the dosage of superovulation drugs, making it the preferred strategy for ART in PCOS patients. Developing an accurate clinical prediction model to help PCOS patients evaluate treatment outcomes before transplantation is of great significance for formulating individualized diagnosis and treatment regimens and improving clinical pregnancy rates. To date, few predictive models for PCOS FET outcomes simultaneously combine metabolic profiles and endometrial Doppler ultrasound indicators, and this study provides exploratory evidence for this research direction.

In this study, 10 key predictive variables were screened through LASSO regression, and the nomogram model was finally constructed based on 7 independent influencing factors (type of infertility, number of subendometrial blood flow branches, endometrial thickness, TC, TG, LDL-C, FPG) and 2 borderline significant indicators with clinical relevance (baseline FSH, AMH), all of which are independent predictive factors for clinical pregnancy in PCOS patients undergoing FET cycles.

Age is a classic predictive factor for ART outcomes. Multiple studies have confirmed that ovarian function declines significantly in women aged ≥35 years, and the clinical pregnancy rate and live birth rate after ART are significantly reduced (29). Similar conclusions were obtained in this study, suggesting that advanced age is an adverse factor for pregnancy in FET cycles of PCOS patients. As a core biomarker of ovarian reserve, AMH can accurately reflect the number of antral follicles and the response to controlled ovarian stimulation. A recent study enrolling 1003 IVF cycles verified that serum AMH was significantly positively correlated with the number of retrieved oocytes and could effectively predict poor ovarian response and high ovarian response (30). In PCOS patients, elevated AMH reflects increased number of small antral follicles and disordered folliculogenesis, which may further affect oocyte quality, endometrial receptivity and final clinical pregnancy. This evidence supports the biological rationality of including AMH as a candidate predictor in the model. As a sensitive biomarker of ovarian reserve, AMH has been widely reported to be associated with reproductive prognosis in PCOS populations. A recent observational study by (31) including 220 PCOS patients undergoing artificial insemination found that patients with middle-range AMH levels (4.08–8.99 ng/mL) tended to have higher pregnancy and live birth rates compared with those with low or high AMH levels, supporting a non-linear relationship between AMH and reproductive outcomes. Consistent with this evidence, AMH was retained in our model as a clinically relevant prognostic indicator, reflecting the ovarian reserve status of PCOS patients and its potential impact on endometrial receptivity and embryo implantation.

There is a close causal relationship between PCOS and obesity. Mendelian randomization studies have confirmed that elevated body fat distribution indicators (BMI, waist-to-hip ratio) are significantly associated with the incidence of PCOS (32). Obesity can adversely affect follicular development, the epigenetic process of embryogenesis, and endometrial receptivity by altering the maternal metabolic environment; meanwhile, excess adipose tissue can aromatize androgens into estrogens, interfering with the hypothalamic-pituitary-ovarian axis function and further aggravating the endocrine disorder of PCOS. PCOS is also a common cause of secondary hyperlipidemia. Studies have shown that compared with age-matched healthy people, patients with PCOS have significantly higher TG and LDL-C levels, and the elevation of LDL-C remains statistically significant after adjusting for BMI. Obesity and dyslipidemia can aggravate all clinical manifestations and reproductive outcome impairments of PCOS, including menstrual disorders, hyperandrogenemia, hyperglycemia, infertility, miscarriage, and pregnancy complications (33). Existing studies have confirmed that abnormal blood lipids can negatively affect the live birth rate of ART in PCOS patients (34). High BMI and abnormal glucose metabolism will increase the risk of clinical miscarriage after IVF in PCOS patients (35), and the mechanism may be related to the damage of obesity and dyslipidemia to oocyte and embryo quality (36, 37). Notably, BMI was compressed to zero by LASSO regression in our study, which does not negate its clinical prognostic importance in PCOS populations. As reported by (31), higher BMI shows a trend toward reduced pregnancy and live birth rates in PCOS patients undergoing ART. The exclusion of BMI in our model is most likely attributable to its collinearity with the included metabolic indicators (TC, TG, LDL-C, FPG); its predictive effect on clinical pregnancy is mainly mediated through metabolic disorders, rather than exerting an independent effect after adjustment for detailed lipid and glucose parameters.

Beyond BMI, several other variables with established clinical relevance were also excluded by LASSO regression. Endometrial preparation protocol (natural vs. artificial cycle) was compressed to zero, likely because after adjusting for endometrial thickness and subendometrial blood flow—which are more direct measures of endometrial receptivity—the specific preparation protocol contributes limited independent predictive information in PCOS patients undergoing FET with high-quality embryos. Type of embryos transferred (cleavage-stage vs. blastocyst) was also excluded. Since all cycles in this study were restricted to morphologically high-quality embryos, the pregnancy outcome difference between cleavage-stage and blastocyst transfers was minimized, reducing its independent predictive signal in the LASSO model. Duration of infertility, endometrial pattern (Type A vs. non-Type A), and number of embryos transferred were similarly compressed to zero, suggesting that their predictive information was largely captured by other variables in the model (e.g., age, infertility type, and endometrial thickness), or that their effects were not sufficiently independent in this specific cohort. Importantly, LASSO exclusion does not imply that these variables are clinically irrelevant—rather, it indicates that their contributions to prediction are largely mediated through, or redundant with, the retained variables in the current model. Future studies with larger and more diverse populations may better delineate the independent roles of these factors.

Medication regimens during endometrial preparation were not individually adjusted for. However, all patients followed the standardized clinical protocols of our center, with dose adjustments primarily guided by endometrial thickness; thus, the confounding effect from regimen variation is likely limited. Endometrial preparation protocol and embryo type were addressed in the LASSO exclusion analysis above. These factors, together with the unmeasured confounders listed below, represent the main sources of residual confounding in this retrospective study.

Meanwhile, lipid peroxidation caused by hyperlipidemia can impair vascular endothelial cell function and alter hemodynamics, thereby affecting endometrial receptivity and pregnancy establishment. In this study, we found that elevated TC, TG, and LDL-C are independent risk factors for clinical pregnancy in FET cycles of PCOS patients, which provides further clinical evidence for the above research conclusions.

This study also confirmed that the number of subendometrial blood flow branches is significantly associated with clinical pregnancy in PCOS patients undergoing FET cycles, and the pregnancy probability is significantly higher when the number of subendometrial blood flow branches is ≥10, which is consistent with the research conclusion of Zhang et al. (26). Angiogenesis is a key link in embryo implantation. The capillary network formed by the uterine artery extending through the myometrium to the endometrial surface can provide sufficient blood supply for embryo implantation and pregnancy maintenance (38). In recent years, Doppler ultrasound technology has been widely used to evaluate hemodynamic parameters related to endometrial receptivity, but the predictive value of endometrial blood flow indicators for clinical pregnancy is still controversial (39): some studies believe that endometrial and subendometrial vascular parameters in IVF cycles cannot effectively predict clinical pregnancy, while studies on patients with thin endometrium in FET cycles have shown that subendometrial blood flow status is significantly positively correlated with embryo implantation and clinical pregnancy (38). The results of this study further confirm that the more terminal arterial branches under the endometrium in FET cycles, the more conducive it is to improving the pregnancy rate, suggesting that the number of subendometrial blood flow branches can be used as an effective ultrasound indicator for evaluating endometrial receptivity.

Since all cycles included in this study were limited to high-quality embryo transfers, the difference in embryo morphological quality was controlled to the maximum extent. At present, there is still controversy about the impact of the type of transferred embryos on FET clinical pregnancy. A large retrospective cohort study by Cameron et al. (40) showed that the cumulative live birth rate of blastocyst transfer was higher than that of cleavage-stage embryo transfer, but the difference was not statistically significant after balancing baseline differences; while a study by Kashi et al. (41) found that the type of transferred embryos was an independent influencing factor for clinical pregnancy in FET cycles, and the pregnancy rate of blastocyst transfer was significantly higher than that of cleavage-stage embryo transfer. This study also found that the clinical pregnancy rate of blastocyst transfer was higher, suggesting that the embryonic development stage may have an important impact on FET outcomes of PCOS patients, and blastocyst transfer can be preferentially selected in clinical practice according to the patient’s condition.

This study represents an exploratory effort to integrate metabolic and ultrasonographic parameters in a single prediction model for FET outcomes in PCOS, whose advantages are reflected in three aspects: First, the scientificity of variable screening. Non-predictive variables were eliminated through LASSO regression (λ=0.02045), which avoided the overfitting problem of traditional multivariate regression. The final 9 variables included (7 independent influencing factors +2 borderline significant indicators) have all passed strict statistical verification. Second, the comprehensiveness of model validation. The AUC of the model was 0.74 and 0.75 in the training and internal validation cohorts, respectively. The calibration curve was in good agreement with the ideal diagonal line, the Hosmer-Lemeshow test indicated no overfitting, and DCA suggested potential net benefit within the threshold range of 0.20–0.80, though this finding should be interpreted cautiously given the modest discriminative ability. The net benefit was superior to the ‘all intervention’ or ‘no intervention’ strategies within this range. Third, the nomogram format allows intuitive scoring and probability reading without complex calculation, which provides convenience for subsequent clinical verification and application after further optimization. Compared with metabolic-only models reported in other studies (though direct comparison was not performed), this model additionally incorporates endometrial blood flow parameters which addresses some limitations of prior models that focused solely on metabolic parameters. The nomogram can be conveniently integrated into routine clinical workflows of reproductive medicine centers. Before FET transplantation, clinicians can collect the required indicators through routine examinations, quickly calculate the predicted probability of clinical pregnancy through the nomogram, and carry out personalized risk stratification for patients. The application of the model does not require additional equipment or complex operations; clinicians can master the scoring method through brief training. For patients with low predicted probability, targeted pre-transplant interventions such as metabolic regulation and endometrial perfusion improvement can be implemented to optimize clinical pregnancy. In the future, we will develop an online web-based calculator based on this model to further improve the convenience of clinical application.

Several important limitations affect the interpretability and generalizability of our model. First, the model explains only approximately 20–25% of outcome variation (estimated Nagelkerke R²), indicating that the majority of factors determining clinical pregnancy remain unmeasured. Second, while all transferred embryos were restricted to morphologically high-quality embryos (graded using Veeck classification for cleavage-stage embryos and Gardner classification for blastocysts) to eliminate morphological confounding, molecular developmental indicators were not collected; embryo developmental potential remains one of the strongest established predictors of implantation success. Third, insulin resistance—a central pathophysiologic feature of PCOS—was captured only by fasting glucose rather than more sensitive measures such as HOMA-IR or fasting insulin, which may independently affect endometrial receptivity and implantation. The absence of these markers is a major limitation, as insulin resistance is mechanistically central to PCOS pathophysiology. Fourth, the optimal cut-off value of 0.6765, determined solely via Youden’ s index in the internal cohort, lacks external population verification and should be recalibrated for different clinical contexts. Therefore, this cut-off value should currently be considered a reference for research-based risk stratification rather than a direct clinical decision threshold. Fifth, the AUC of 0.74-0.75, while statistically significant, is below the 0.80 threshold typically considered sufficient for standalone clinical decision support.

Supplementary methodological limitations include: ① the retrospective design introduces potential information bias and residual confounding from unmeasured factors (e.g., medication adherence during endometrial preparation, lifestyle factors, psychological status, and inflammatory markers); ② several predictors (e.g., age) were dichotomized for nomogram simplification, potentially losing subtle nonlinear relationships with outcomes, though sensitivity analysis treating age as a continuous variable yielded similar results (AUC 0.75); ③ interaction effects between metabolic and endometrial factors were not modeled due to limited statistical power; ④ the split-sample internal validation, while methodologically acceptable, does not guarantee generalizability to other populations or settings, and multi-center external validation is urgently needed; ⑤ all findings are population-level and cannot be directly extrapolated to individual patients without clinical judgment. Therefore, this model should be viewed as a preliminary tool for research-based risk stratification rather than a formal clinical decision aid. Future prospective studies incorporating standardized insulin resistance indices, embryonic molecular assessments, and multi-center external validation are needed to optimize predictive performance before any consideration of clinical implementation.

In this single-center retrospective study, a nomogram incorporating metabolic biomarkers (TC, TG, LDL-C, FPG) and endometrial ultrasonographic parameters (thickness, subendometrial blood flow) achieved modest discrimination (AUC 0.74-0.75) for predicting clinical pregnancy in PCOS patients undergoing FET. While these predictors may be independently associated with outcomes, the model explains only a portion of outcome variation. Critical missing predictors—including standardized molecular embryo metrics and dedicated insulin resistance markers—limit the model’s current clinical utility. After external validation and further refinement, such a model might eventually support risk stratification, but at present, it should be considered exploratory and hypothesis-generating.

## Data Availability

The raw data supporting the conclusions of this article will be made available by the authors, without undue reservation.
